# Longitudinal change of serum AIM2 levels after aneurysmal subarachnoid hemorrhage and its prognostic significance: a two-center prospective cohort study

**DOI:** 10.1038/s41598-024-61185-1

**Published:** 2024-05-07

**Authors:** Ziyin Chen, Shengdong Zou, Hao Shan, Jin Liu, Da Tian, Xiaoyu Wu, Quan Du, Xiaoqiao Dong, Dingbo Yang

**Affiliations:** 1 The Fourth School of Clinical Medicine, Zhejiang Chinese Medical University, No. 548 Binwen Road, Hangzhou, 310053 China; 2 Department of Neurosurgery, The Sixth Affiliated Hospital of Wenzhou Medical University, No. 15 Dazhong Road, Lishui, 323000 China; 3 Department of Neurosurgery, Affiliated Hangzhou First People’s Hospital, Westlake University School of Medicine, No. 261 Huansha Road, Hangzhou, 310006 China

**Keywords:** Absent in melanoma 2, Biomarkers, Aneurysm, Subarachnoid hemorrhage, Delayed cerebral ischemia, Prognosis, Severity, Biomarkers, Risk factors

## Abstract

Absent in melanoma 2 (AIM2) is implicated in neuroinflammation. Here, we explored the prognostic significance of serum AIM2 in human aneurysmal subarachnoid hemorrhage (aSAH). We conducted a consecutive enrollment of 127 patients, 56 of whom agreed with blood-drawings not only at admission but also at days 1, 2, 3, 5, 7 and 10 days after aSAH. Serum AIM2 levels of patients and 56 healthy controls were measured. Disease severity was assessed using the modified Fisher scale (mFisher) and World Federation of Neurological Surgeons Scale (WFNS). Neurological outcome at poststroke 90 days was evaluated via the modified Rankin Scale (mRS). Univariate analysis and multivariate analysis were sequentially done to ascertain relationship between serum AIM2 levels, severity, delayed cerebral ischemia (DCI) and 90-day poor prognosis (mRS scores of 3–6). Patients, in comparison to controls, had a significant elevation of serum AIM2 levels at admission and at days 1, 2, 3, 5, 7 and 10 days after aSAH, with the highest levels at days 1, 2, 3 and 5. AIM2 levels were independently correlated with WFNS scores and mFisher scores. Significantly higher serum AIM2 levels were detected in patients with a poor prognosis than in those with a good prognosis, as well as in patients with DCI than in those without DCI. Moreover, serum AIM2 levels independently predicted a poor prognosis and DCI, and were linearly correlated with their risks. Using subgroup analysis, there were no significant interactions between serum AIM2 levels and age, gender, hypertension and so on. There were substantially high predictive abilities of serum AIM2 for poor prognosis and DCI under the receiver operating characteristic curve. The combination models of DCI and poor prognosis, in which serum AIM2, WFNS scores and mFisher scores were incorporated, showed higher discriminatory efficiencies than anyone of the preceding three variables. Moreover, the models are delineated using the nomogram, and performed well under the calibration curve and decision curve. Serum AIM2 levels, with a substantial enhancement during early phase after aSAH, are closely related to bleeding severity, poor 90-day prognosis and DCI of patients, substantializing serum AIM2 as a potential prognostic biomarker of aSAH.

## Introduction

Non-traumatic subarachnoid hemorrhage (SAH) is a life-threatening and highly fatal neurological condition, with aneurysmal rupture accounting for approximately 85% of such cases^1^. Aneurysmal SAH (aSAH) is characterized by a rapid onset, high disability and high mortality rate. Early brain injury involves inflammatory reaction, oxidative stress and apoptosis, and has been believably accepted as a very important mechanism, which leads to neuronal injury^2,3^. One of the most common and serious complications is delayed cerebral ischemia (DCI), which may be mainly caused by cerebral vasospasm and increases the risk of poor prognosis in patients with aSAH^4^. It is a crucial step to predict DCI and even a poor prognosis during the clinical management of aSAH. Biomarkers can assist in assessing severity and predicting prognosis. In recent decades, measurements of circulating biomarkers, such as complement component 1q, A20, S100B and copeptin, have been increasingly noted with respect to their prognostic predictive values in aSAH^5–8^.

Inflammasomes are a group of multimeric protein complexes that can protect host against infectious agents and physiological aberration^9^. Absent in melanoma 2 (AIM2) is an inflammasome receptor that can be activated in response to double-stranded DNA^10^. AIM2 engages caspase-1 to catalyze proteolytic cleavage of pro-interleukin-1β and pro-interleukin-18 and drive pyroptosis, thereby inducing inflammation and cellular death^11,12^. AIM2 is mainly expressed in glial cells^13^. Its expressions could be greatly up-regulated in animal brain tissues at some pathological situations, such as Alzheimer’s disease, autoimmune encephalomyelitis, ischemic stroke and SAH^13–16^. AIM2 could damage neurons during ischemic and hemorrhagic injury via increasing neuroinflammation and oxidative stress after experimental SAH, hypoxic-ischemic brain injury, middle cerebral artery occlusion and traumatic brain injury^17–20^. Noteworthily, acute ischemic stroke patients had significantly higher levels of plasma AIM2 than healthy controls; moreover, plasma AIM2 levels were intimately correlated with severity and 90-day poor prognosis after stroke^21^, indicating that AIM2 may be a biomarker of acute brain injury. Here, serum AIM2 was measured and its prognostic role was investigated in aSAH.

## Methods and materials

### Participants

In this prospective observational study, we consecutively recruited patients with aSAH at the Affiliated Hangzhou First People’s Hospital, Westlake University School of Medicine and the Sixth Affiliated Hospital of Wenzhou Medical University between April 2020 and December 2022. Our study included patients with first-time nontraumatic SAH. We specified the following eligibility criteria: (1) patients must be 18 years of age or older; (2) SAH was verified using head computed tomography (CT) scans; (3) aneurysm diagnosis must be confirmed through computed tomography angiography (CTA) or digital subtraction angiography (DSA); (4) patients should be admitted to the hospital within 24 h of symptom onset; (5) ruptured intracranial aneurysms should be repaired within 48 h of admission through surgical clipping or interventional embolization. Exclusion criteria included: (1) severe diseases in other organs; (2) previous neurological diseases, such as ischemic or hemorrhagic stroke, myasthenia gravis, severe traumatic brain injury, moyamoya disease, arteriovenous malformation, or intracranial neoplasm; (3) suspected pseudoaneurysm or aneurysm rebleeding; (4) loss to follow-up, insufficient information, rejection to participation, or missing samples. The study was performed in accordance with the Declaration of Helsinki and its amends. The study protocol was approved by the ethical committees at the Affiliated Hangzhou First People's Hospital, Westlake University School of Medicine (No. 058-01) and the Sixth Affiliated Hospital of Wenzhou Medical University (No. 2020-001). And written informed consent was obtained from the patients' relatives and controls themselves.

### Variables

The data we collected included (1) demographical information, such as age and gender; (2) the presence of underlying chronic diseases, such as hypertension, diabetes and hyperlipidemia; (3) medications, such as antiplatelet agents, anticoagulants and hormones; (4) unhealthy lifestyle habits, such as cigarette smoking and alcohol consumption. To assess the patient's health status and determine appropriate treatment options, non-invasive techniques were used to measure vital signs, such as blood pressure, respiratory rate, heart rate and blood oxygen saturation. The modified Fisher (mFisher) scale and the World Federation of Neurosurgical Societies (WFNS) scale, which provide standardized ways to assess and classify the severity of SAH, were employed as the two indicators for evaluating the radiological and clinical severity of the hemorrhage. CT scans were made to identify intraventricular hemorrhage and acute hydrocephalus, and further head CTA or DSA was performed to determine the location, size and shape of intracranial aneurysms. When determining whether neurosurgical clipping or endovascular intervention was selected as a treatment option for securing aneurysms, the following factors were cautiously taken into consideration, including aneurysm characteristics, patient preferences, overall patient conditions, presence of intracerebral bleeding, intracranial pressure, etc.

The following specific criteria were referred to assess the occurrence of DCI: (1) clinical deterioration (such as new focal deficit, decrease in level of consciousness, or both), and/or (2) detection of new infarction on head CT scans that was not visible on admission or in the early postoperative period and cannot be attributed to any other cause through clinical evaluation, brain imaging, and appropriate laboratory tests^22^. Follow-up on neurologic function was done until 90 days after the injury, and the prognosis was evaluated using the modified Rankin Scale (mRS). The scores of 3–6 on the mRS indicated a poor prognosis^23^.

### Immune analysis

At admission, blood samples were taken from patients and some routine laboratory parameters (e.g., blood white cell count, platelet count, hemoglobin level, and blood glucose) were measured by conventional methods. For the sake of measuring serum AIM2 levels, blood samples of controls were acquired at their entry into study; all patients underwent blood-collections at admission and a portion of patients had blood-obtainments at other multiple time-points, namely, at days 1, 3, 5, 7 and 10. To measure serum AIM2 levels, venous blood samples were spun for 15 min at 3000×*g* and serum was isolated. And then, an aliquot of serum sample was taken and stored promptly at − 80 °C until testing. Then, serum AIM2 levels were quantified in duplicate using an enzyme-linked immunosorbent assay kit (Shanghai Jianglai Biotechnology Co., Ltd, China; Catalogue number, JL49566) according to the manufacturer's instructions. The detection range of this kit was from 0.156 ng/ml to 10 pg/ml, with intra-assay precision of ≤ 10% and inter-assay precision of ≤ 10%. The final analysis was performed using the average value of the two measurements. All measurements were carried out by the same technician, who was inaccessible to clinical materials.

### Statistical analysis

The data were analyzed using the SPSS 25.0 (BMI Software Inc., USA). Graphs were drawn using the GraphPad Prism 9.0 (GraphPad Software, Inc., Boston, MA, USA) and R software (version 4.2.4; https://www.r-project.org). Qualitative variables were expressed as frequency (percentage), and normally and non-normally distributed continuous variables were expressed as mean (standard deviation, SD) and median (interquartile range) respectively. The comparisons of various variables between the two groups were performed using the Chi-square test or Fisher's exact test for qualitative data, and the Mann–Whitney *U* test or independent *t*-test for quantitative data. The Kruskal–Wallis test was used for multiple-group comparisons of quantitative data. The Spearman correlation coefficient was used to calculate the bivariate correlation between serum AIM2 levels and other variables, such as WFNS score and mFisher score. After adjusting for other confounding factors which were significant on univariate analysis (P < 0.05), multivariable linear regression correlation analysis was performed to determine the variables, which were independently correlated with serum AIM2 levels. To determine the factors, which were independently associated with 90-day adverse prognosis and DCI, the binary logistic regression model was established, in which the significant variables on the univariate analysis (P < 0.05) were included. The results were expressed as odds ratios (ORs) and 95% confidence intervals (CIs). Receiver operating characteristic (ROC) curves were used to study the predictive value of serum AIM2 levels for DCI and 90-day adverse prognosis in patients. The area under the curve (AUC) was calculated. The Youden's J method was applied to identify the optimal threshold of serum AIM2 levels to further distinguish patients at risk of DCI or 90-day adverse prognosis, with corresponding sensitivity and specificity values. The independent predictors of DCI or adverse prognosis were incorporated to form prediction model. And the model was described using the nomogram, and was assessed using the calibration curve, decision curve and ROC curve. A two-tailed P-value less than 0.05 was considered statistically significant.

## Results

### Patient selection and characteristics

**Figure 1:**
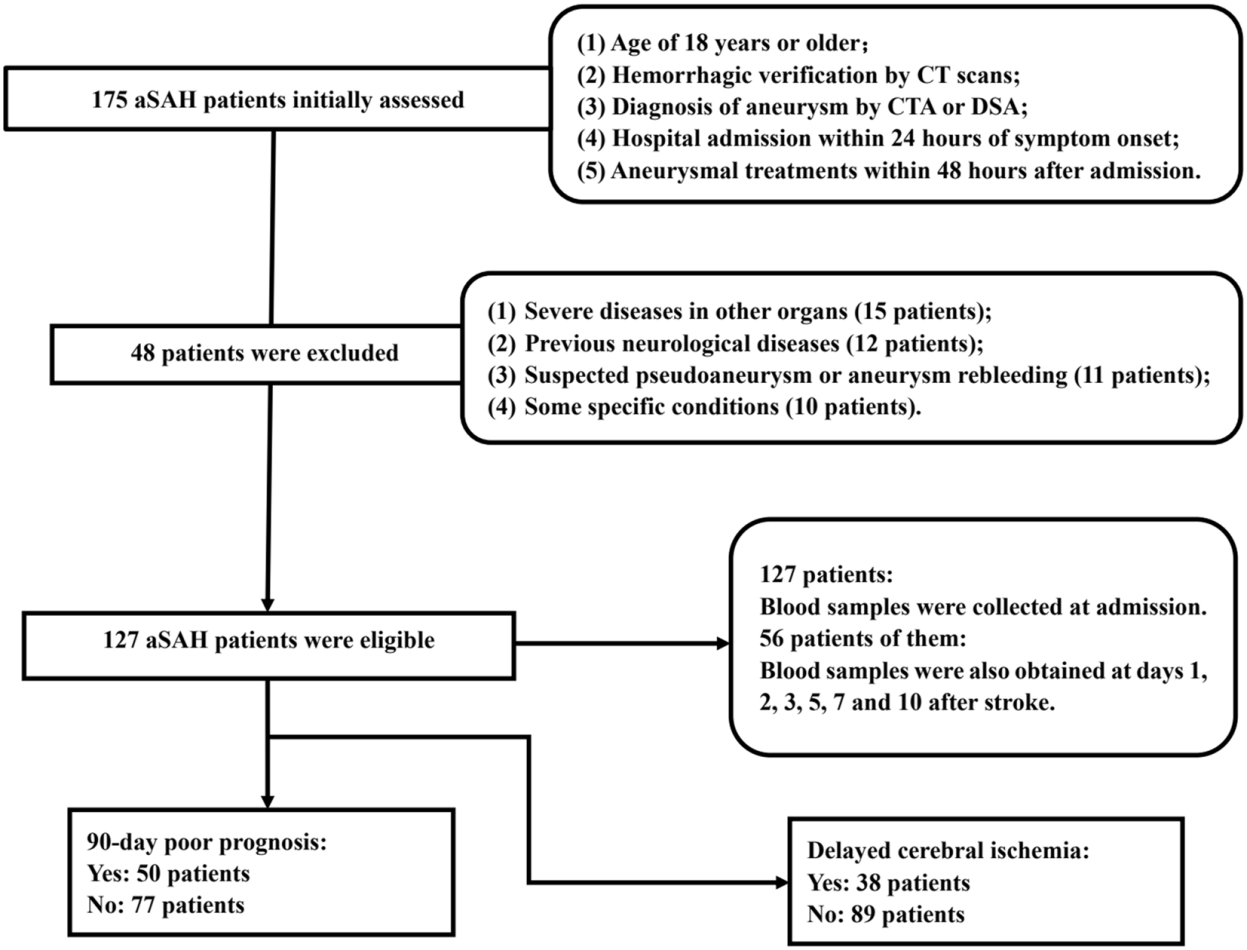
Flowing-chart for selecting eligible patients with aneurysmal subarachnoid hemorrhage. Initially, 175 patients with aneurysmal subarachnoid hemorrhage were assessed and then 48 patients were excluded according to the exclusion criteria. Ultimately, 127 patients were analyzed. *aSAH* aneurysmal subarachnoid hemorrhage.

In this prospective cohort study, a total of 175 patients with first-ever SAH underwent initial evaluation. Among them, 48 patients were excluded because of the reasons outlined in Fig. 1. Of the finally enrolled 127 patients, 56 consented for blood-collection at multiple time points and constituted a special group for uncovering dynamic change of serum AIM2 levels after stroke. Among these 56 patients, 29 were females and 27 were males, 16 smoked cigarettes and 12 drank alcohol. Their mean age was 53.4 years (SD, 10.2 years). A total of 56 healthy controls were recruited. Among these 56 healthy controls, 34 were females and 22 were males, 13 smoked cigarettes and 15 drank alcohol. Their mean age was 51.7 years (SD, 10.6 years). There were no significant differences in age, sex ratio, smoking and drinking between those 56 aSAH patients and 56 healthy controls (all P > 0.05). As shown in Table 1. There were no significant differences in clinical, radiological and biochemical indexes between all 127 patients and those 56 patients (all P > 0.05).

**Table 1:** Comparisons of baseline characteristics between all patients and those volunteers with aneurysmal subarachnoid hemorrhage.

Components	All patients	Voluntary patients	P value
Number	127	56	
Gender (male/female)	56/71	27/29	0.606
Age (years)	52.3 ± 10.4	53.4 ± 10.2	0.531
Cigarette smoking	35 (27.6%)	16 (28.6%)	0.888
Alcohol consumption	31 (24.4%)	12 (21.4%)	0.661
Hypertension	91 (71.7%)	39 (69.6%)	0.782
Diabetes mellitus	11 (8.7%)	2 (3.6%)	0.356
World Federation of Neurological Surgeons Scale scores	3 (2–4)	3 (2–4)	0.682
Modified Fisher scores	3 (2–3)	3 (2–3)	0.379
Aneurysmal position (posterior/anterior circulation)	32/95	16/40	0.632
Aneurysmal shape (cystic/others)	102/26	43/13	0.676
Aneurysmal diameter (< 10 mm/≥ 10 mm)	68/59	24/32	0.183
Methods for securing aneurysms (clipping/endovascular intervention)	58/69	26/30	0.324
Acute hydrocephalus	16 (12.6%)	6 (10.7%)	0.718
Intraventricular bleeding	25 (19.7%)	11 (19.6%)	0.995
External ventricular drain	15 (11.8%)	6 (10.7%)	0.803
Admission time (h)	9.0 (5.1–13.5)	9.5 (5.1–14.5)	0.736
Blood-collection time (h)	10.0 (5.7–14.0)	10.7 (5.8–15.5)	0.770
Systolic arterial pressure (mmHg)	149.2 ± 23.8	148.7 ± 26.4	0.909
Diastolic arterial pressure (mmHg)	90.0 ± 16.0	88.6 ± 16.7	0.584
Blood glucose levels (mmol/l)	10.6 (7.5–14.9)	9.9 (7.4–14.6)	0.659
Serum C-reactive protein levels (mg/l)	8.6 (4.3–14.6)	9.4 (5.2–16.6)	0.319

Quantitative data were reported as medians with 25th–75th percentiles or the mean ± standard deviation as appropriate. Qualitative data were presented as counts (proportions). Intergroup comparisons of various variables were performed using the χ^2^ test or Fisher’s exact test for qualitative data, and *t* test or Mann–Whitney *U* test for quantitative data.

### Temporal change of serum AIM2 levels after aSAH and its correlation with severity

**Figure 2:**
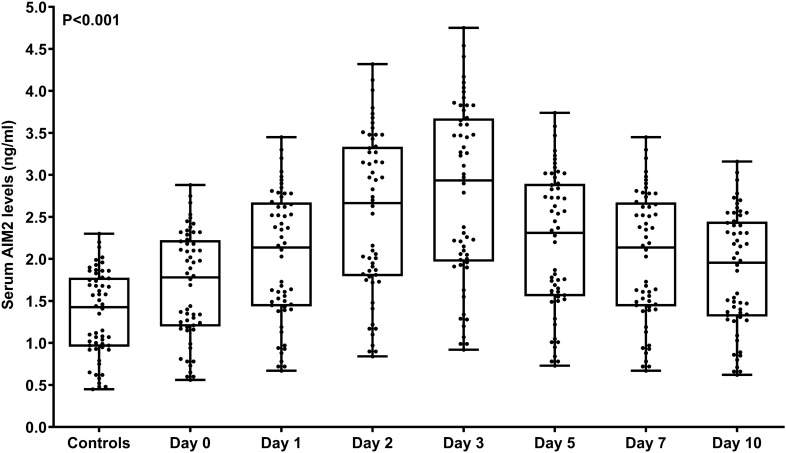
Differences in terms of serum absent in melanoma 2 levels between controls and patients with aneurysmal subarachnoid hemorrhage. Serum absent in melanoma 2 levels were significantly higher in patients than in controls (P < 0.001), with the highest levels in patients at days 1, 2, 3 and 5. AIM2 denotes absent in melanoma 2.

**Figure 3:**
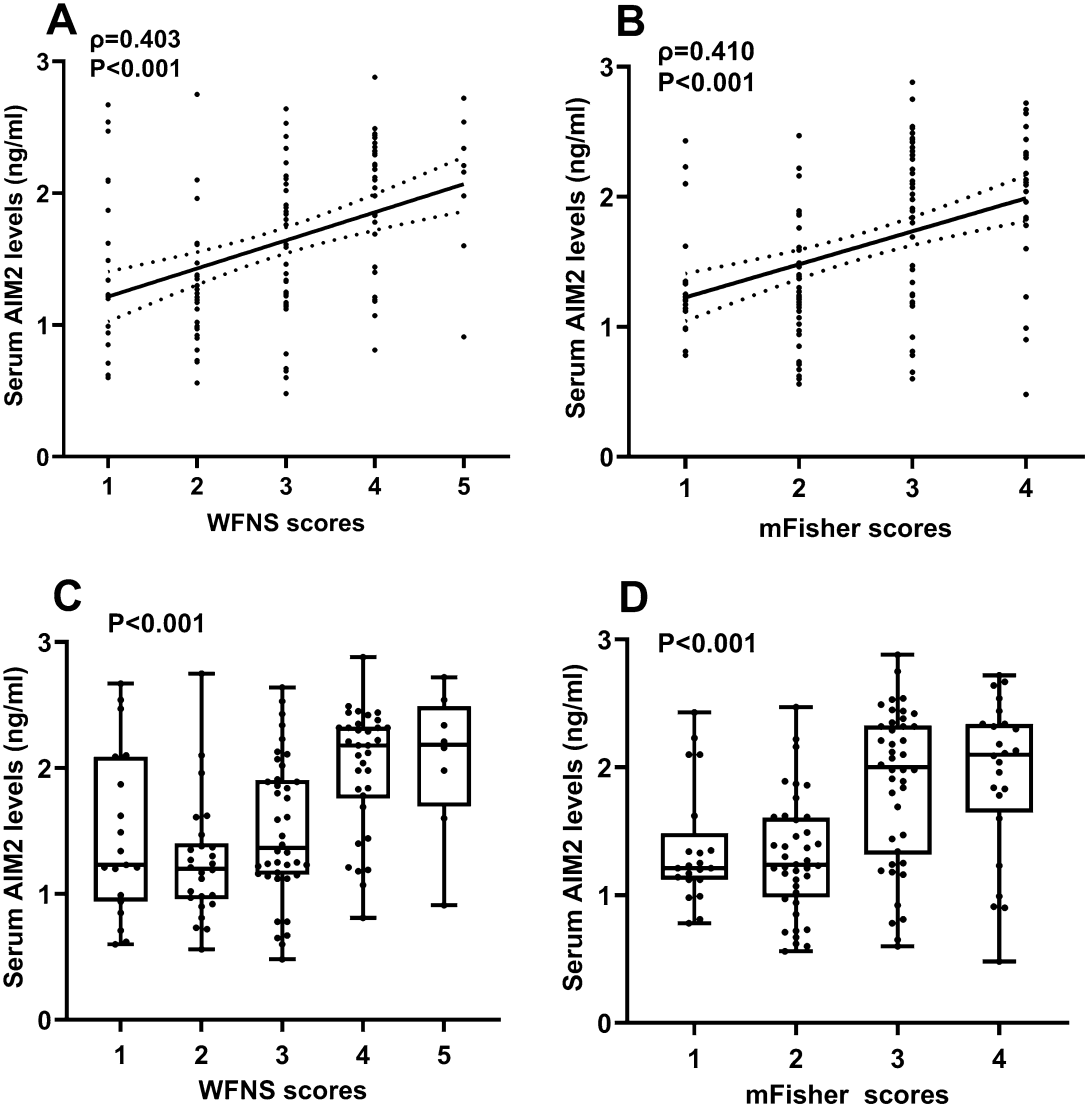
Relationship between serum absent in melanoma 2 levels and severity after aneurysmal subarachnoid hemorrhage. Serum absent in melanoma 2 levels were intimately positively correlated with World Federation of Neurosurgical Societies Scale scores (P < 0.001; **A**) and modified Fisher scores (P < 0.001; **B**), as well as the levels were significantly elevated with increasing with World Federation of Neurosurgical Societies Scale scores (P < 0.001; **C**) and modified Fisher scores (P < 0.001; **D**). *AIM2* absent in melanoma 2, *WFNS* World Federation of Neurosurgical Societies, *mFisher* modified Fisher.

Compared with the control group, the serum AIM2 levels of those 56 patients increased significantly on admission, the highest levels appeared at days 1, 2, 3 and 5, and the serum AIM2 levels decreased slowly at day 7 until day 10. Serum AIM2 levels of patients were substantially higher during 10 days than those of controls (P < 0.001; Fig. 2). Serum AIM2 levels of all 127 patients, ranging from 0.48 to 2.88 ng/ml with a median value of 1.59 ng/ml (25th–75th percentiles, 1.17–2.16 ng/ml), were equivalent to those of those 56 patients (P > 0.05). Also, there were non-statistically significant differences in terms of serum AIM2 levels between all 127 patients and those 56 patients (P > 0.05). Serum AIM2 levels were substantially positively correlated with WFNS scores (P < 0.001; Fig. 3A) and mFisher scores (P < 0.001; Fig. 3B), as well as were significantly increased in order of WFNS scores (P < 0.001; Fig. 3C) and mFisher scores (P < 0.001; Fig. 3D). In Table 2, serum AIM2 levels showed a close correlation with other variables, including intraventricular bleeding, blood glucose levels and serum C-reactive protein levels (all P < 0.05). When adding the above variables to the multivariate linear regression model, serum AIM2 levels were independently associated with WFNS scores (β, 0.125; 95% CI, 0.030–0.220; VIF, 0.747; P = 0.010) and mFisher scores (β, 0.124; 95% CI, 0.006–0.242; VIF, 0.666; P = 0.039).

**Table 2:** Bivariate correlation analysis between serum absent in melanoma 2 levels and other variables in 127 aneurysmal subarachnoid hemorrhage patients.

Components	ρ	P value
Gender (male/female)	0.071	0.428
Age (years)	0.025	0.780
Cigarette smoking	0.017	0.851
Alcohol consumption	0.018	0.845
Hypertension	0.158	0.075
Diabetes mellitus	0.064	0.474
World Federation of Neurological Surgeons Scale scores	0.419	< 0.001
Modified Fisher scores	0.413	< 0.001
Aneurysmal position (posterior/anterior circulation)	0.009	0.923
Aneurysmal shape (cystic/others)	0.072	0.420
Aneurysmal diameter (< 10 mm/≥ 10 mm)	− 0.064	0.478
Methods for securing aneurysms (surgical clipping/endovascular intervention)	− 0.056	0.528
Acute hydrocephalus	− 0.023	0.795
Intraventricular bleeding	0.238	0.007
External ventricular drain	0.045	0.613
Admission time (h)	0.135	0.130
Blood-collection time (h)	0.053	0.553
Systolic arterial pressure (mmHg)	0.134	0.133
Diastolic arterial pressure (mmHg)	0.026	0.775
Blood glucose levels (mmol/l)	0.254	0.004
Serum C-reactive protein levels (mg/l)	0.254	0.004

Bivariate correlations were completed using Spearman’s rank correlation test.

### Relationship between serum AIM2 levels and 90-day functional outcome after aSAH

Among those patients who consented for blood-collections at multiple time-points, cases with development of poor prognosis (mRS scores of 3–6) accounted for 42.9% (24/56). In this portion of patients, serum AIM2 levels at days 1, 2, 3, 5, 7 and 10 days after aSAH had AUCs at 0.734 (95% CI, 0.628–0.883), 0.778 (95% CI, 0.658–0.902), 0.750 (95% CI, 0.602–0.865), 0.710 (95% CI, 0.559–0.833), 0.668 (95% CI, 0.515–0.798) and 0.653 (95% CI, 0.500–0.786) respectively. As compared to those at admission (AUC, 0.682; 95% CI, 0.530–0.810), they had non-statistically significant AUCs for predicting 90-day poor prognosis (all P > 0.05).

**Figure 4:**
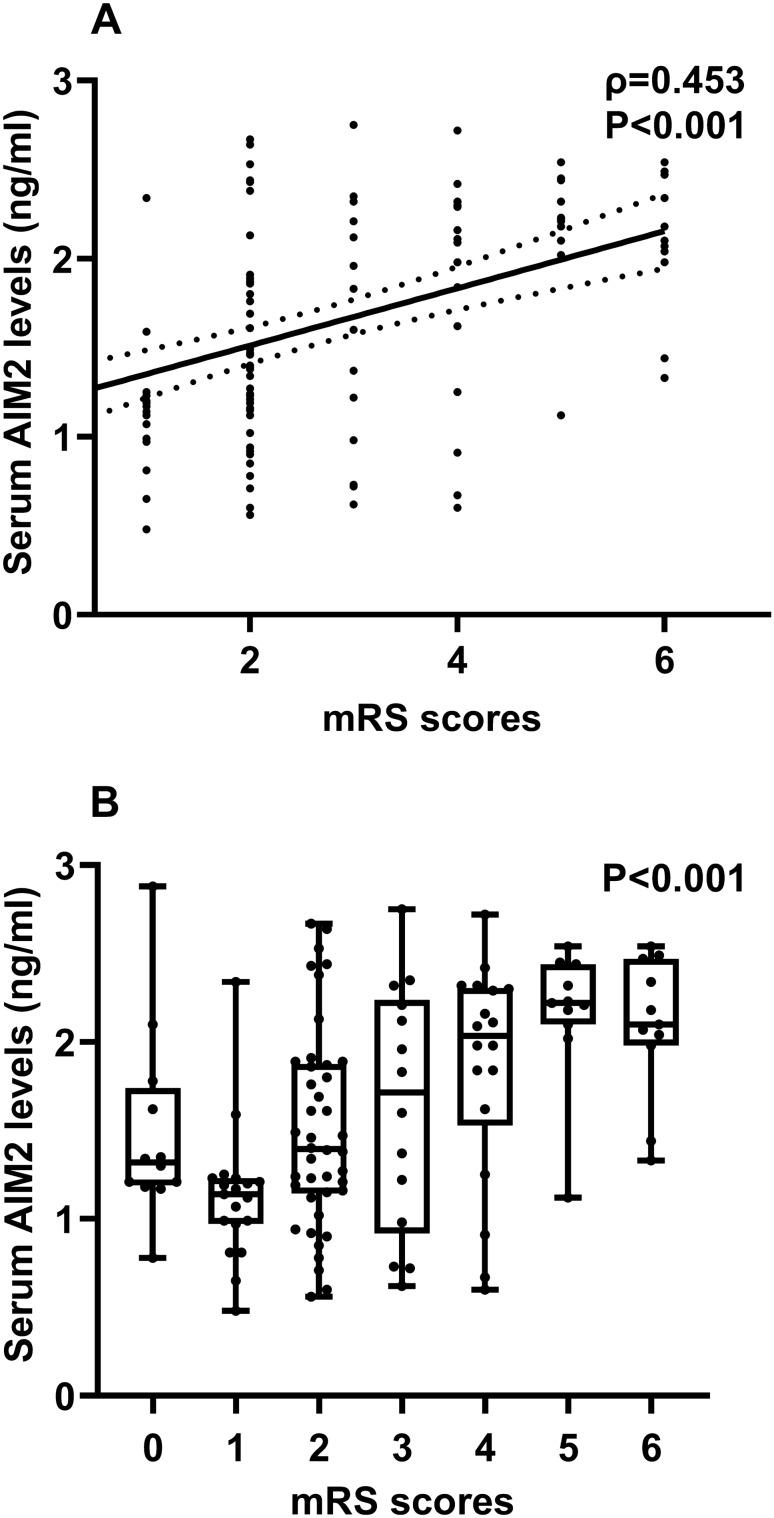
Relationship between serum absent in melanoma 2 levels and modified Rankin scale scores after aneurysmal subarachnoid hemorrhage. Serum absent in melanoma 2 levels were tightly positively correlated with modified Rankin Scale scores (P < 0.001; **A**) and the levels were pronouncedly elevated with raising modified Rankin Scale scores (P < 0.001; **B**). *AIM2* absent in melanoma 2, *mRS* modified Rankin Scale.

**Figure 5:**
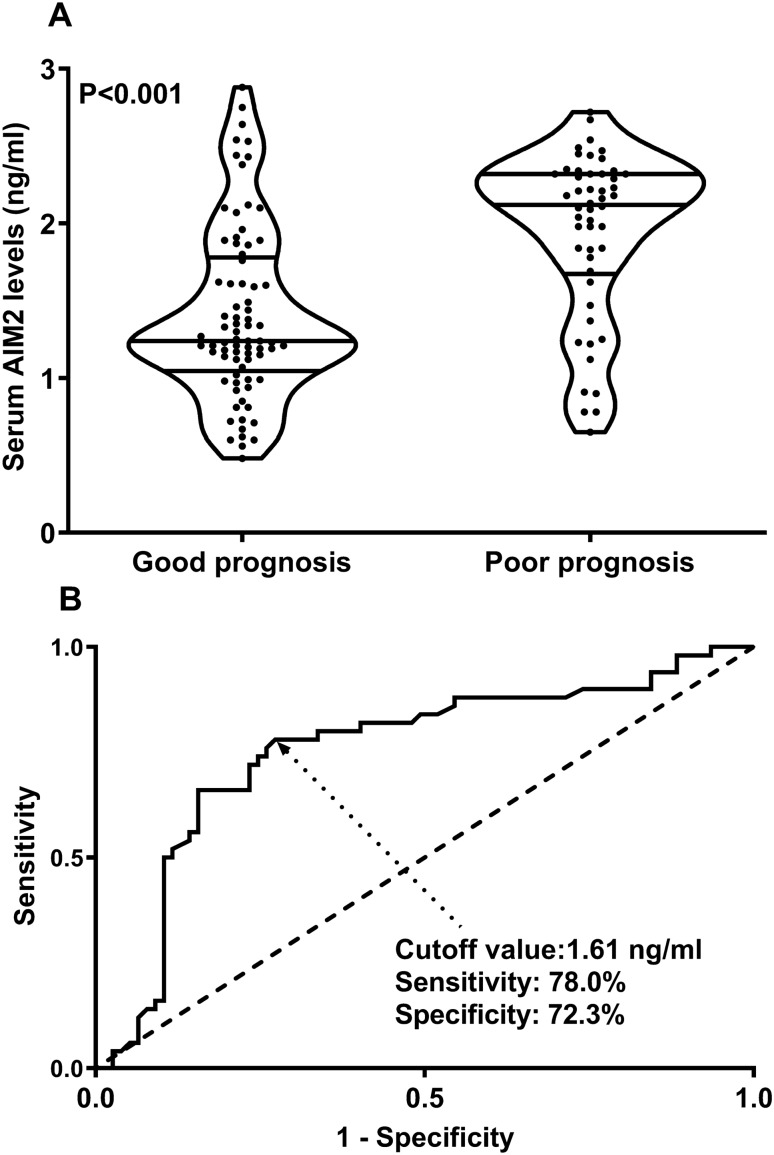
Serum absent in melanoma 2 levels and poor prognosis at 90 days after aneurysmal subarachnoid hemorrhage. Serum absent in melanoma 2 levels were markedly higher in patients with poor prognosis than in those with good prognosis (P < 0.001; **A**) and substantially predicted poor prognosis at 90 days after aneurysmal subarachnoid hemorrhage (**B**). *AIM2* absent in melanoma 2.

**Figure 6:**
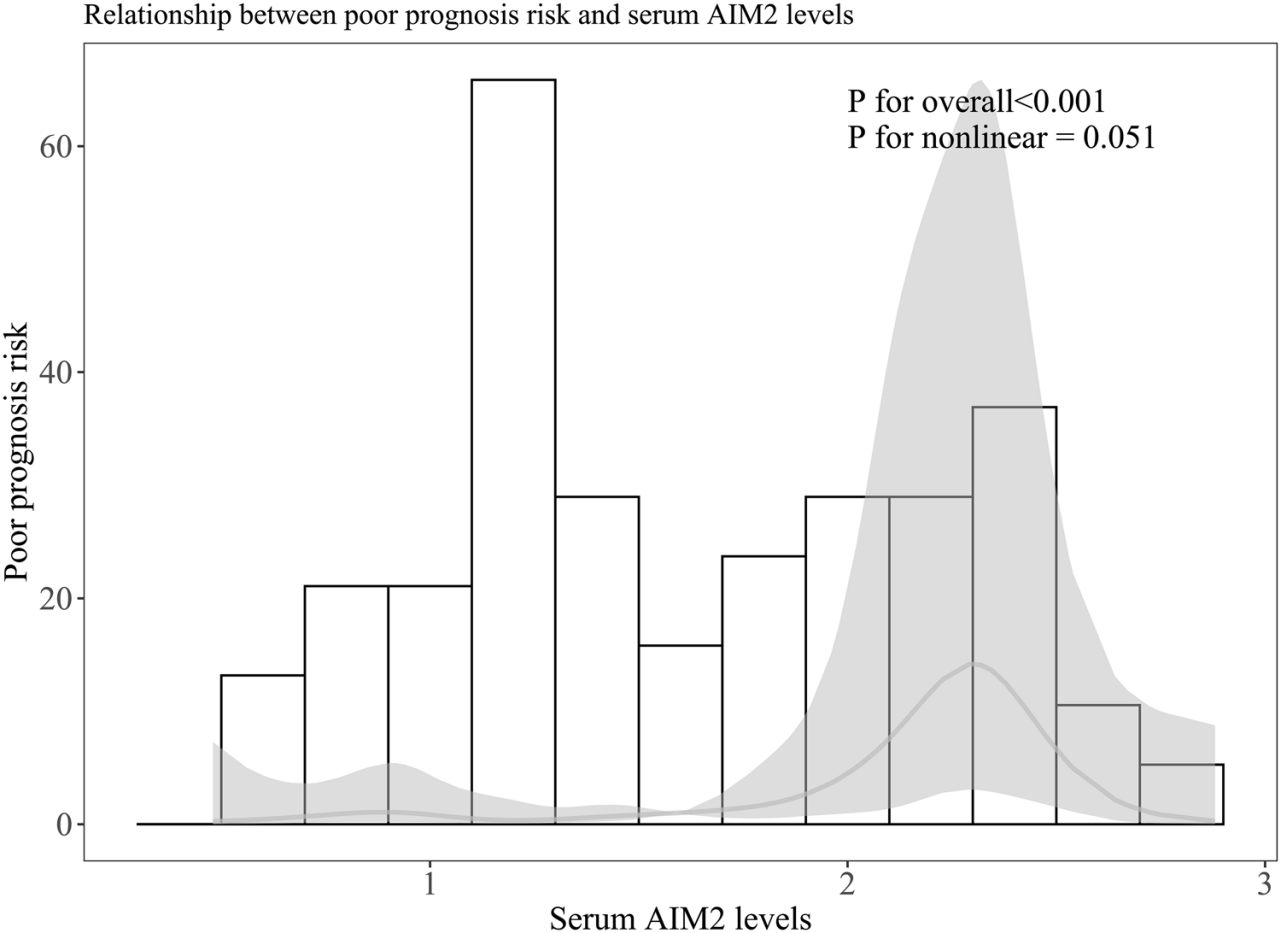
Restricted cubic spline showing presence of linear relationship between serum absent in melanoma 2 levels and risk of poor prognosis at 90 days after aneurysmal subarachnoid hemorrhage. Serum absent in melanoma 2 levels were obviously linearly correlated with poor prognosis risk at 90 days after aneurysmal subarachnoid hemorrhage (P > 0.05). *AIM2* absent in melanoma 2.

Among all 127 patients, 50 patients had a poor prognosis at 90 days after the onset of aSAH. Serum AIM2 levels were significantly positively correlated with mRS scores (P < 0.001; Fig. 4A), were substantially elevated in order of mRS scores (P < 0.001; Fig. 4B). The concentration of serum AIM2 in patients with poor prognosis was significantly higher than that in patients with good prognosis. (P < 0.001; Fig. 5A). Under the ROC curve, associated criterion was 1.61 ng/ml using the Youden method. The sensitivity of serum AIM2 level to predict the risk of 90-day poor prognosis was 78.0%, and the specificity was 72.3% (Fig. 5B). In addition, restricted cubic spline analysis showed a linear association of serum AIM2 levels with the risk of poor prognosis at 90 days (Fig. 6, P > 0.05).

**Figure 7:**
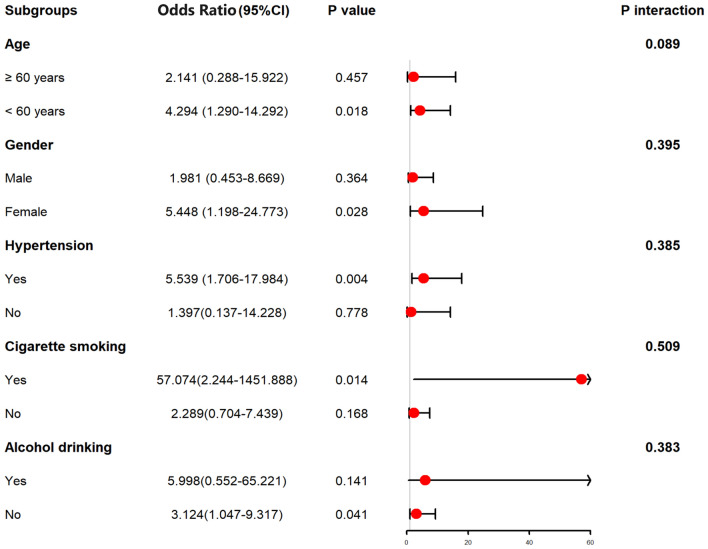
Subgroup analysis showing interaction between serum absent in melanoma 2 levels and other variables in poor prognosis prediction of aneurysmal subarachnoid hemorrhage. No significant interactions were found between serum absent in melanoma 2 levels and other variables, such as age, gender, hypertension and so on (all P > 0.05). *95% CI* 95% confidence interval.

As listed in Table 3, as opposed to patients with good prognosis, those presenting whit poor prognosis had significantly higher proportion of serum AIM2 levels (P < 0.001), displayed substantially higher percentage of intraventricular hemorrhage (P < 0.05), and tended to exhibit markedly higher blood glucose levels (P < 0.05), serum C-reactive protein levels (P < 0.05), WFNS scores (P < 0.001) and mFisher scores (P < 0.001). All the abovementioned significant variables were forced into the multivariate model, and it was found that WFNS score (OR, 1.591; 95% CI, 1.022–2.493; P = 0.040), mFisher score (OR, 1.822; 95% CI, 1.063–3.134; P = 0.030) and serum AIM2 levels (OR, 2.389; 95% CI, 1.079–5.290; P = 0.032) independently predicted the occurrence of poor prognosis at 90 days after hemorrhagic stroke. We further analyzed interactions of serum AIM2 levels on age, sex, hypertension, smoking and drinking, and found that no significantly statistical interactions existed (all P > 0.05; Fig. 7).

**Table 3:** Differences of baseline characteristics by clinical outcome at 90 days after aneurysmal subarachnoid hemorrhage.

Components	Poor outcome	Good outcome	P value
Gender (male/female)	23/27	33/44	0.727
Age (years)	54.4 ± 10.3	51.0 ± 10.4	0.077
Cigarette smoking	12 (24.0%)	23 (29.9%)	0.469
Alcohol consumption	12 (24.0%)	19 (24.7%)	0.931
Hypertension	37 (74.0%)	54 (70.1%)	0.636
Diabetes mellitus	6 (12.0%)	5 (6.5%)	0.450
World Federation of Neurological Surgeons Scale scores	4 (3–4)	3 (2–3)	< 0.001
Modified Fisher scores	3 (3–4)	2 (2–3)	< 0.001
Aneurysmal position (posterior/anterior circulation)	14/36	18/59	0.558
Aneurysmal shape (cystic/others)	40/10	61/16	0.915
Aneurysmal diameter (< 10 mm/≥ 10 mm)	25/25	34/43	0.519
Methods for securing aneurysms (surgical clipping/endovascular intervention)	26/24	32/45	0.248
Acute hydrocephalus	5 (10.0%)	11 (14.3%)	0.477
Intraventricular bleeding	16 (32.0%)	9 (11.7%)	0.005
External ventricular drain	8 (16.0%)	7 (9.1%)	0.239
Admission time after stroke (h)	10.5 (5.5–14.2)	8.1 (4.8–12.5)	0.330
Blood-collection time (h)	10.7 (5.9–14.6)	9.5 (4.6–13.8)	0.988
Systolic arterial pressure (mmHg)	146.6 ± 24.7	150.8 ± 23.3	0.333
Diastolic arterial pressure (mmHg)	88.5 ± 15.4	91.1 ± 16.3	0.374
Blood glucose levels (mmol/l)	12.4 (7.4–17.0)	10.1 (7.6–12.5)	0.040
Serum C-reactive protein levels (mg/l)	12.3 (7.2–17.2)	6.1 (2.6–13.2)	< 0.001
Serum absent in melanoma 2 levels (mg/l)	2.1 (1.7–2.3)	1.2 (1.1–1.8)	< 0.001

Quantitative data were reported as medians with 25th–75th percentiles or the mean ± standard deviation as appropriate. Qualitative data were presented as counts (proportions). Intergroup comparisons of various variables were performed using the χ^2^ test or Fisher’s exact test for qualitative data, and Mann–Whitney *U* test for quantitative data.

**Figure 8:**
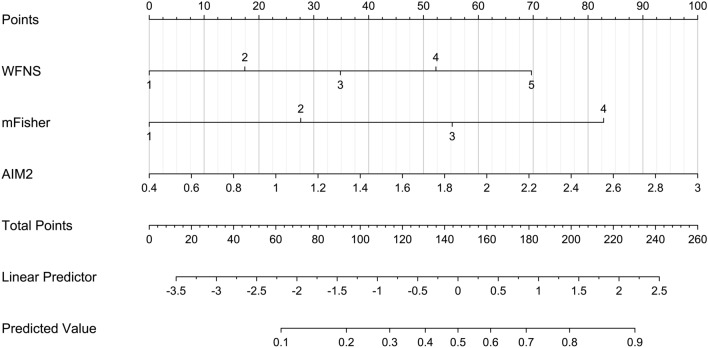
Nomogram displaying poor prognosis prediction model at 90 days after aneurysmal subarachnoid hemorrhage. The model was composed of serum absent in melanoma 2 levels, World Federation of Neurosurgical Societies Scale scores and modified Fisher scores, and was used to predict poor prognosis at 90 days after aneurysmal subarachnoid hemorrhage. AIM2 denotes absent in melanoma 2; WFNS, World Federation of Neurosurgical Societies; mFisher, modified Fisher.

**Figure 9:**
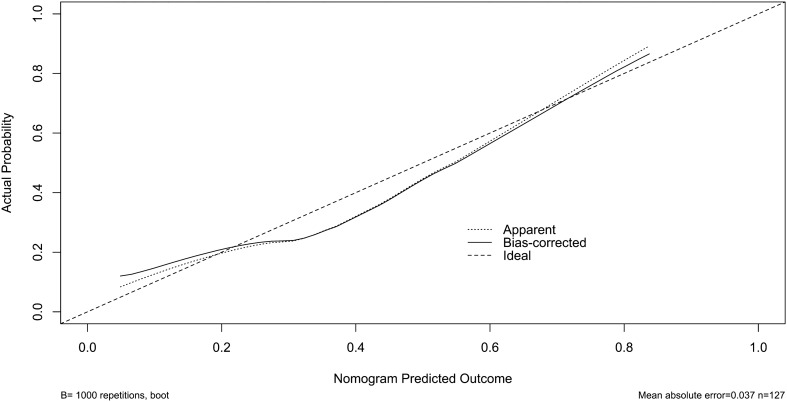
Calibration curve exhibiting stability of poor prognosis prediction model at 90 days after aneurysmal subarachnoid hemorrhage. The model was composed of serum absent in melanoma 2 levels, World Federation of Neurosurgical Societies Scale scores and modified Fisher scores, and was comparatively stable to predict poor prognosis at 90 days after aneurysmal subarachnoid hemorrhage.

**Figure 10:**
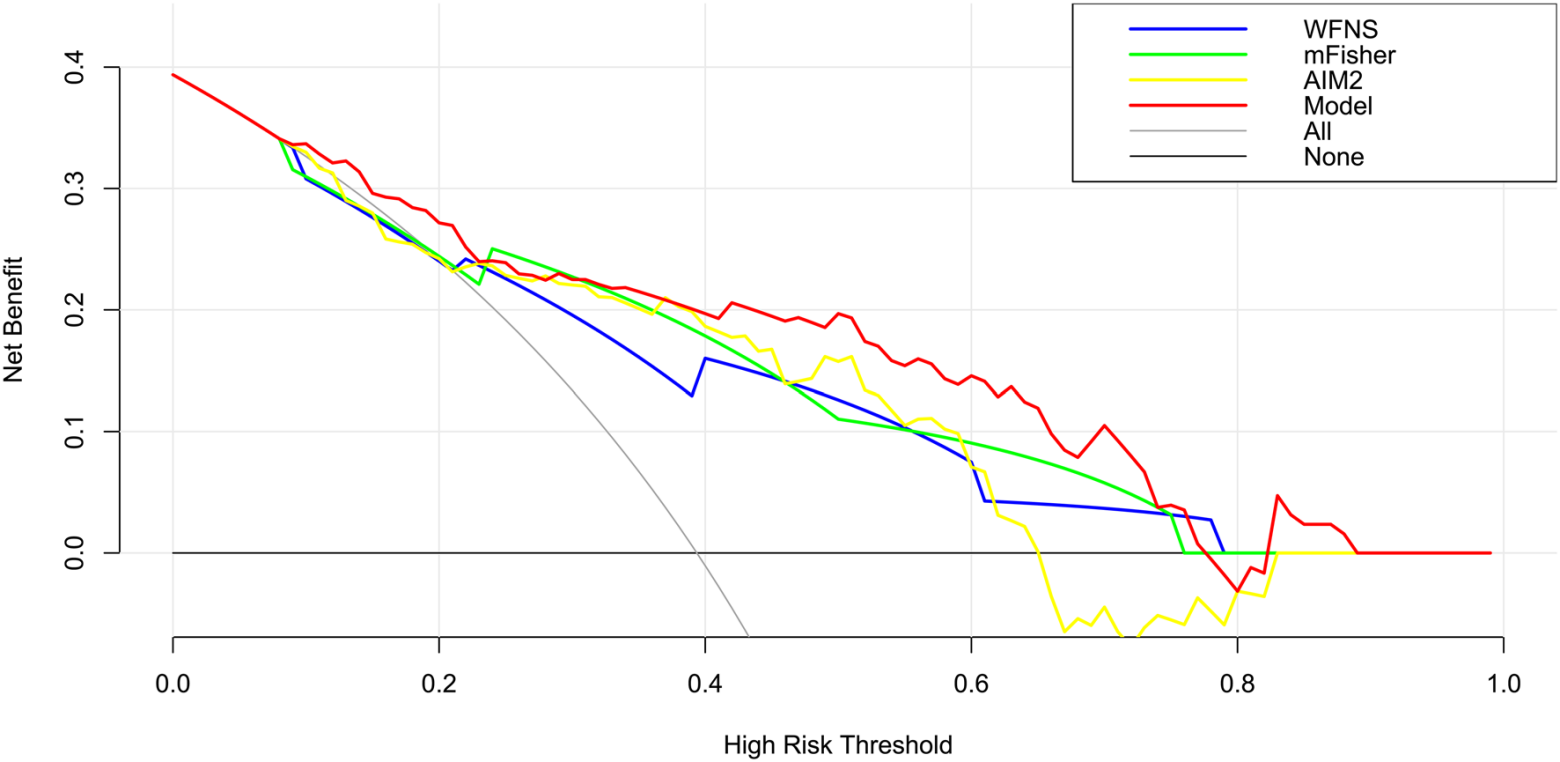
Decision curve depicting clinical effectiveness of poor prognosis prediction model at 90 days after aneurysmal subarachnoid hemorrhage. The model was composed of serum absent in melanoma 2 levels, World Federation of Neurosurgical Societies Scale scores and modified Fisher scores, and was of clinical efficiency to predict poor prognosis at 90 days after aneurysmal subarachnoid hemorrhage. *AIM2* absent in melanoma 2, *WFNS* World Federation of Neurosurgical Societies, *mFisher* modified Fisher.

**Figure 11:**
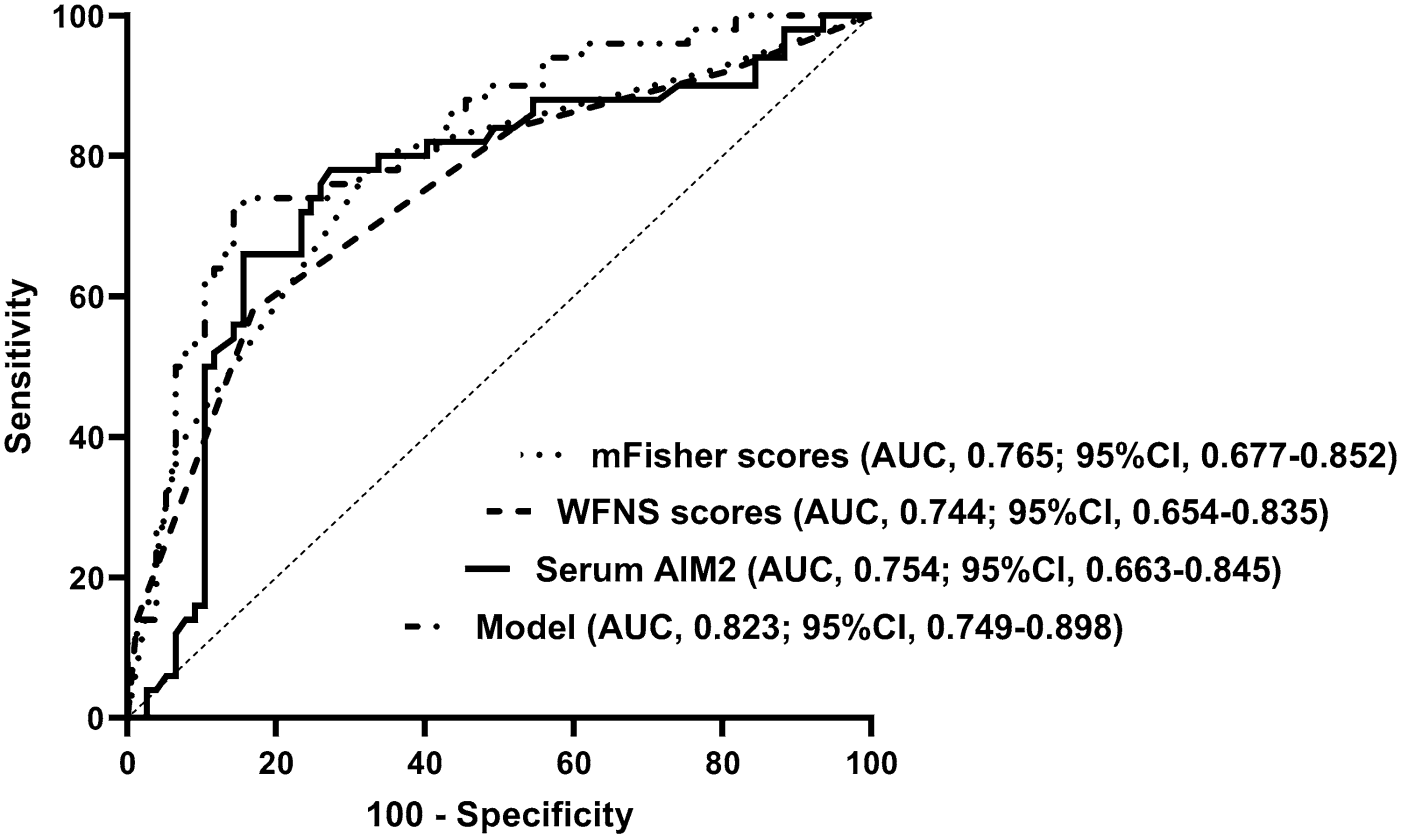
Receiver operating characteristic curve portraying predictive ability of poor prognosis prediction model at 90 days after aneurysmal subarachnoid hemorrhage. The model was composed of serum absent in melanoma 2 levels, World Federation of Neurosurgical Societies Scale scores and modified Fisher scores, and was used to predict poor prognosis at 90 days after aneurysmal subarachnoid hemorrhage, with significantly higher predictive capability than World Federation of Neurosurgical Societies Scale scores, modified Fisher scores and serum absent in melanoma 2 levels (all P < 0.05). *AIM2* absent in melanoma 2, *WFNS* World Federation of Neurosurgical Societies, *mFisher* modified Fisher, *AUC* area under curve, *95% CI* 95% confidence interval.

We established a model of serum AIM2 combined with WFNS score and mFisher score. The model was visually described using a nomogram (Fig. 8). Using calibration curve, the model was comparatively stable (Fig. 9). Using decision curve analysis, the model had comparatively high benefit (Fig. 10). In Fig. 11, serum AIM2 levels effectively distinguished patients at risk of poor prognosis, with AUC > 0.75; the predictive ability of serum AIM2 level was similar to those of mFisher score (P > 0.05) and WFNS score (P > 0.05); and the model had significantly higher predictive capability than that of WFNS score, mFisher score and serum AIM2 level alone (all P < 0.05).

### Relationship between serum AIM2 levels and DCI after aSAH

As for predictive ability for DCI, AUCs of serum AIM2 levels at 1, 2, 3, 5, 7 and 10 days after stroke were 0.758 (0.634–0.887), 0.762 (0.615–0.874), 0.770 (0.588–0.854), 0.682 (0.530–0.810), 0.665 (0.513–0.796) and 0.661 (0.508–0.792) respectively. Their AUCs were equivalent to that of admission serum AIM2 levels (AUC, 0.719; 95% CI, 0.569–0.840) in this group of 56 patients with aSAH (all P > 0.05).

**Figure 12:**
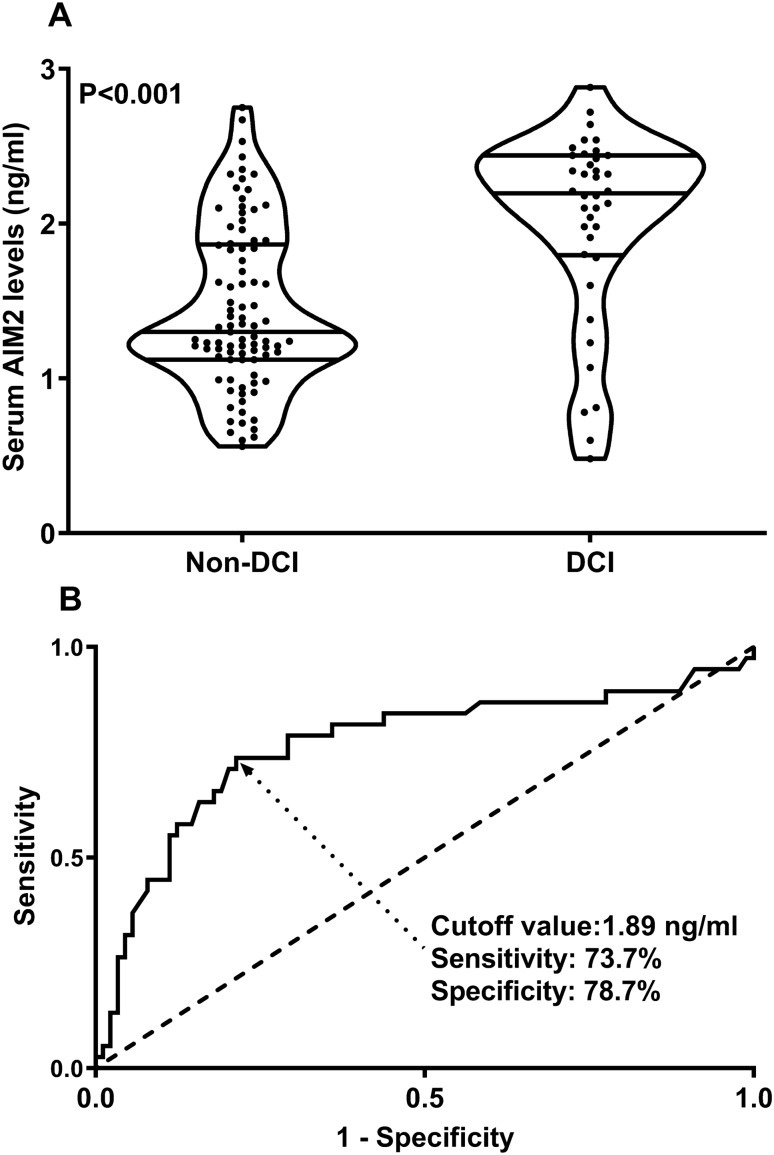
Serum absent in melanoma 2 levels and delayed cerebral ischemia after aneurysmal subarachnoid hemorrhage. Serum absent in melanoma 2 levels were markedly higher in patients with delayed cerebral ischemia than in those without (P < 0.001; **A**) and substantially predicted delayed cerebral ischemia after aneurysmal subarachnoid hemorrhage (**B**). *AIM2* absent in melanoma 2, *DCI* delayed cerebral ischemia.

**Figure 13:**
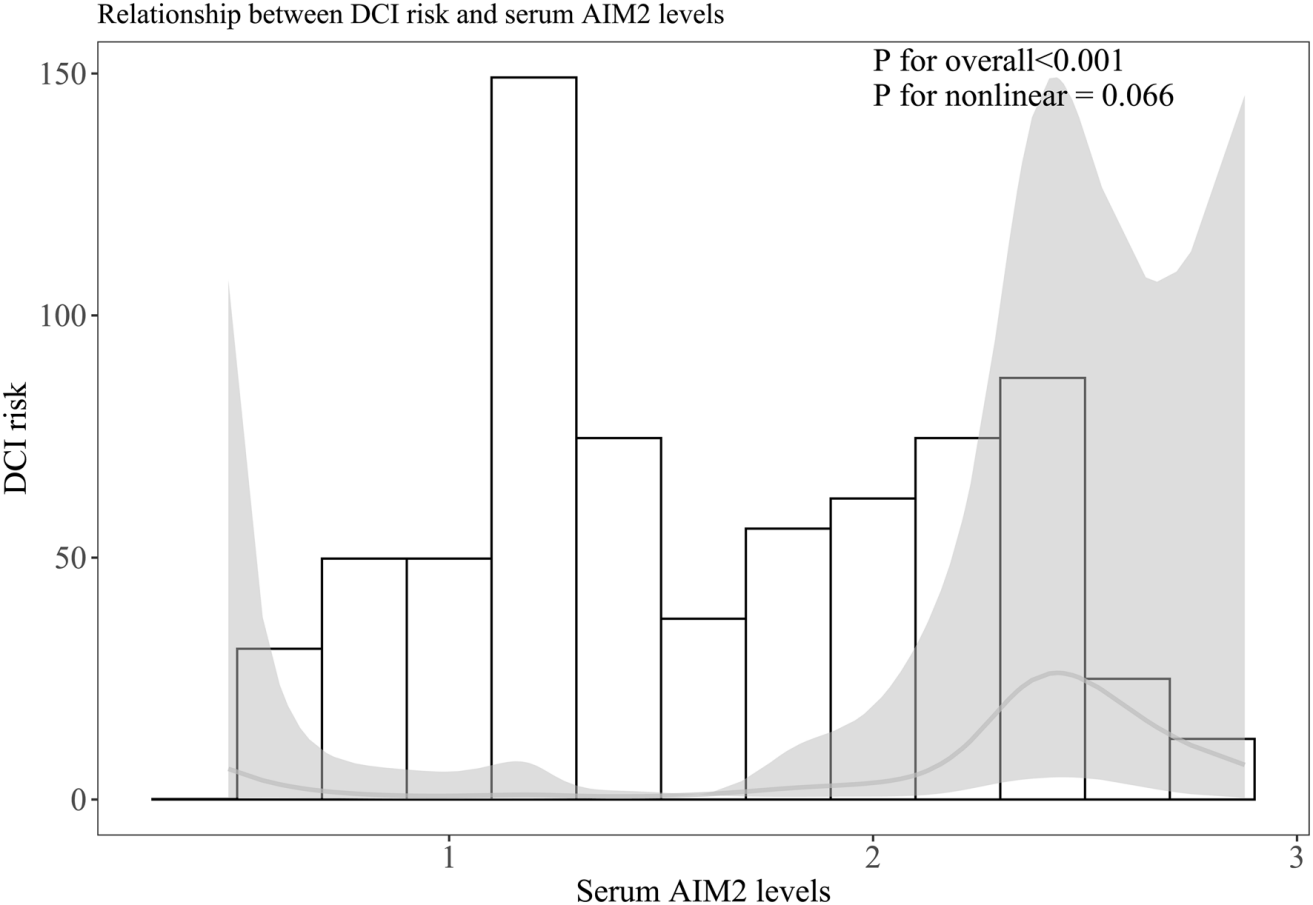
Restricted cubic spline showing presence of linear relationship between serum absent in melanoma 2 levels and risk of delayed cerebral ischemia after aneurysmal subarachnoid hemorrhage. Serum absent in melanoma 2 levels were obviously linearly correlated with delayed cerebral ischemia risk after aneurysmal subarachnoid hemorrhage (P > 0.05). *AIM2* absent in melanoma 2, *DCI* delayed cerebral ischemia.

Among all 127 patients, a total of 38 patients suffered from DCI. Serum AIM2 levels in patients with DCI was significantly higher than that in patients without DCI (Fig. 12A). The sensitivity of serum AIM2 levels to predict the occurrence of DCI was 73.7% and the specificity was 78.7%. Using the Youden method, the associated criterion was 1.89 ng/ml (Fig. 12B). The restricted cubic spline analysis showed a linear association between serum AIM2 levels and DCI risk (Fig. 13, P > 0.05).

**Figure 14:**
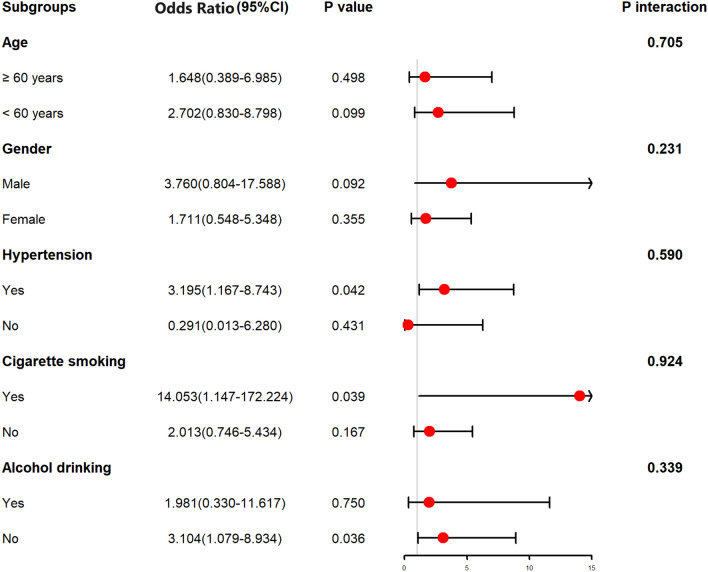
Subgroup analysis showing interaction between serum absent in melanoma 2 levels and other variables in prediction of delayed cerebral ischemia after aneurysmal subarachnoid hemorrhage. No significant interactions were found between serum absent in melanoma 2 levels and other variables, such as age, gender, hypertension and so on (all P > 0.05). *95% CI* 95% confidence interval.

In Table 4, in contrast to patients without development of DCI, those with suffering of DCI had significantly elevated serum AIM2 levels (P < 0.001), as well as exhibited substantially raised blood glucose levels (P < 0.01), intraventricular bleeding (P < 0.05), WFNS scores (P < 0.001) and modified Fisher scores (P < 0.001). Using multivariate analysis, WFNS score (OR, 1.693; 95% CI, 1.043–2.741; P = 0.033), mFisher score (OR, 2.109; 95% CI, 1.144–3.873; P = 0.017) and serum AIM2 levels (OR, 2.640; 95% CI, 1.101–6.329; P = 0.030) were independently associated with DCI risk after aSAH. The subgroup analysis of serum AIM2 level and DCI showed that there were no significantly different interactions between serum AIM2 levels and other variables, such as age, gender, hypertension, smoking, and alcohol drinking (Fig. 14).

**Table 4:** Differences of baseline characteristics across delayed cerebral ischemia after aneurysmal subarachnoid hemorrhage.

Components	Delayed cerebral ischemia		P value
	Presence	Absence	
Gender (male/female)	20/18	36/53	0.205
Age (years)	54.6 ± 9.5	51.4 ± 10.7	0.094
Cigarette smoking	9 (23.7%)	26 (29.2%)	0.523
Alcohol consumption	12 (31.6%)	19 (21.3%)	0.219
Hypertension	31 (81.6%)	60 (67.4%)	0.105
Diabetes mellitus	6 (15.8%)	5 (5.6%)	0.128
World Federation of Neurological Surgeons Scale scores	4 (3–4)	3 (2–3)	< 0.001
Modified Fisher scores	3 (3–4)	2 (2–3)	< 0.001
Aneurysmal position (posterior/anterior circulation)	14/24	18/71	0.080
Aneurysmal shape (cystic/others)	32/6	69/20	0.393
Aneurysmal diameter (< 10 mm/≥ 10 mm)	21/17	47/42	0.800
Methods for securing aneurysms (surgical clipping/endovascular intervention)	15/23	43/46	0.360
Acute hydrocephalus	5 (13.2%)	11 (12.4%)	1.000
Intraventricular bleeding	12 (31.6%)	13 (14.6%)	0.028
External ventricular drain	6 (15.8%)	9 (10.1%)	0.544
Admission time after stroke (h)	11.6 (5.9–15.4)	8.5 (5.0–12.5)	0.054
Blood-collection time (h)	11.4 (7.1–15.6)	9.2 (4.7–13.9)	0.245
Systolic arterial pressure (mmHg)	157.2 ± 25.1	144.0 ± 22.5	0.406
Diastolic arterial pressure (mmHg)	93.2 ± 15.7	88.7 ± 16.0	0.140
Blood glucose levels (mmol/l)	14.8 (9.7–16.7)	9.5 (7.4–12.4)	0.001
Serum C-reactive protein levels (mg/l)	10.1 (4.7–15.8)	7.3 (4.0–14.0)	0.496
Serum absent in melanoma 2 level (ng/ml)	2.2 (1.8–2.4)	1.3 (1.1–1.9)	< 0.001

Qualitative data were shown as count (proportion). Quantitative data were summarized as mean + standard deviation or median (percentiles 25th–75th) as appropriate. Intergroup comparisons of parameters were done using the Student *t* test, Mann–Whitney test, Fisher’s exact test or χ^2^ test as appropriate.

**Figure 15:**
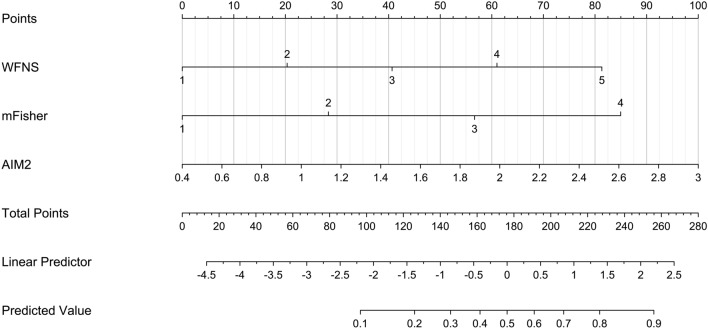
Nomogram displaying delayed cerebral ischemia prediction model after aneurysmal subarachnoid hemorrhage. The model was composed of serum absent in melanoma 2 levels, World Federation of Neurosurgical Societies Scale scores and modified Fisher scores, and was used to predict delayed cerebral ischemia after aneurysmal subarachnoid hemorrhage. *AIM2* absent in melanoma 2, *WFNS* World Federation of Neurosurgical Societies, *mFisher* modified Fisher.

**Figure 16:**
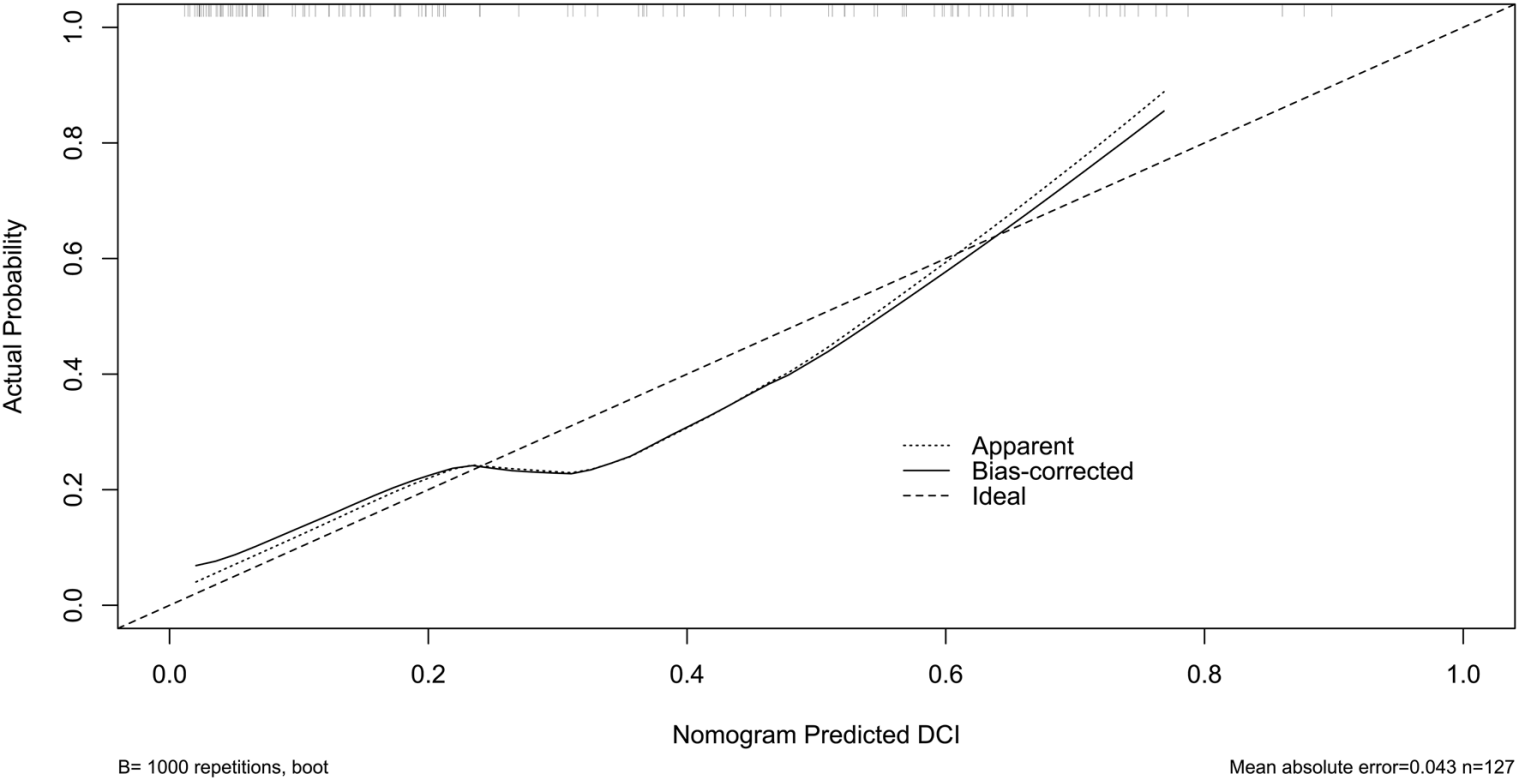
Calibration curve exhibiting stability of delayed cerebral ischemia prediction model after aneurysmal subarachnoid hemorrhage. The model was composed of serum absent in melanoma 2 levels, World Federation of Neurosurgical Societies Scale scores and modified Fisher scores, and was comparatively stable to predict delayed cerebral ischemia after aneurysmal subarachnoid hemorrhage. *DCI* delayed cerebral ischemia.

**Figure 17:**
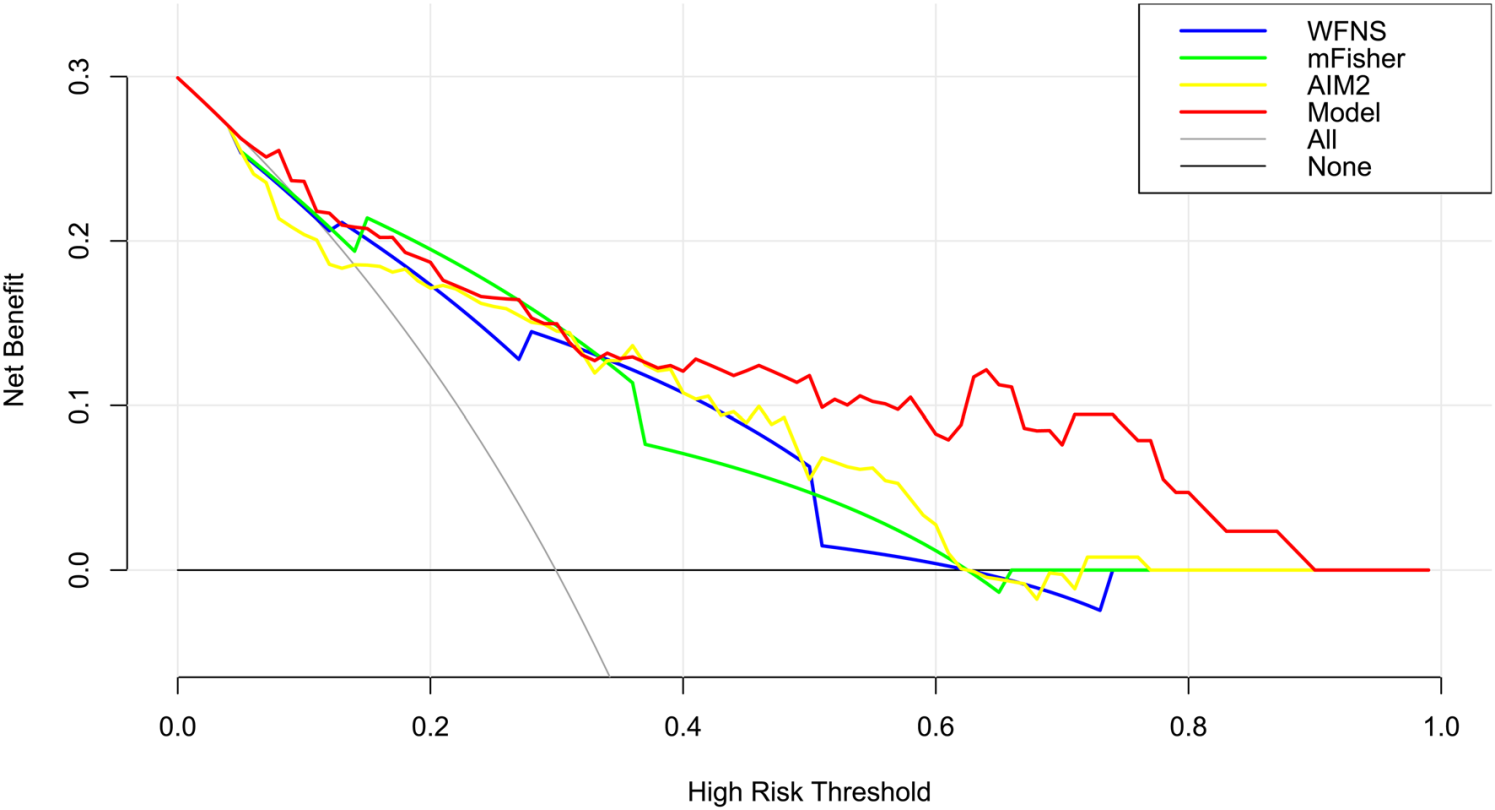
Decision curve depicting clinical effectiveness of delayed cerebral ischemia prediction model after aneurysmal subarachnoid hemorrhage. The model was composed of serum absent in melanoma 2 levels, World Federation of Neurosurgical Societies Scale scores and modified Fisher scores, and was of clinical efficiency to predict delayed cerebral ischemia after aneurysmal subarachnoid hemorrhage. *AIM2* absent in melanoma 2, *WFNS* World Federation of Neurosurgical Societies, *mFisher* modified Fisher.

**Figure 18:**
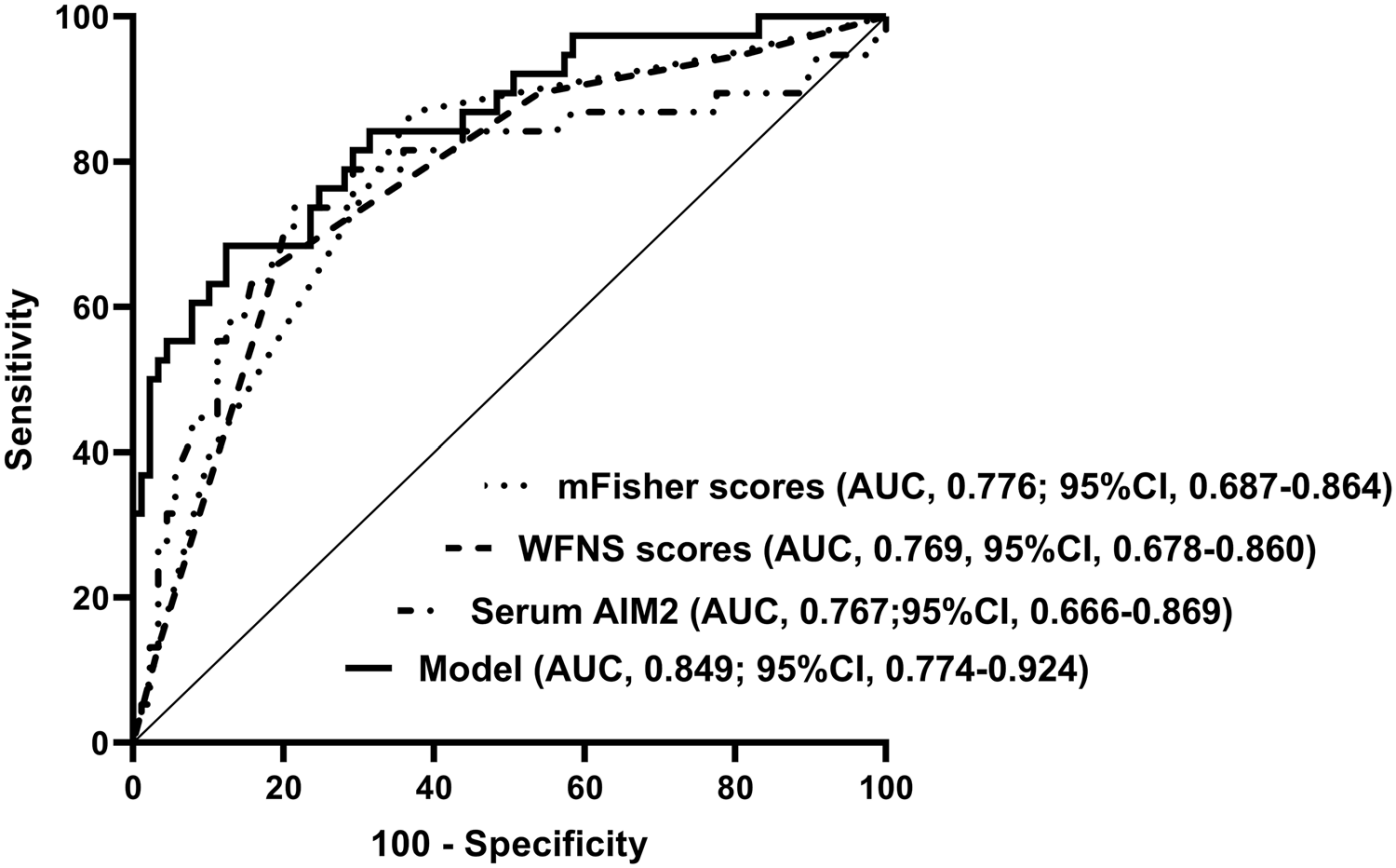
Receiver operating characteristic curve portraying predictive ability of delayed cerebral ischemia prediction model after aneurysmal subarachnoid hemorrhage. The model was composed of serum absent in melanoma 2 levels, World Federation of Neurosurgical Societies Scale scores and modified Fisher scores, and was used to predict delayed cerebral ischemia after aneurysmal subarachnoid hemorrhage, with significantly higher predictive capability than World Federation of Neurosurgical Societies Scale scores, modified Fisher scores and serum absent in melanoma 2 levels (all P < 0.05). *AIM2* absent in melanoma 2, *WFNS* World Federation of Neurosurgical Societies, *mFisher* modified Fisher, *AUC* area under curve, *95% CI* 95% confidence interval.

We also established a model of serum AIM2 combined with WFNS score and mFisher score. The model, which was delineated using nomogram (Fig. 15), was of relative stability (Fig. 16). The prediction models had comparatively high benefit by using decision curve analysis (Fig. 17). As outlined in Fig. 18, serum AIM2 levels were effective in predicting the occurrence of DCI after aSAH (AUC > 0.75); interestingly, serum AIM2 levels had similar predictive ability compared with the mFisher score (P > 0.05) and WFNS score (P > 0.05); combination model, in which serum AIM2, WFNS score and mFisher score were merged, had AUC at 0.849; and the predictive power of the combined model was found to be significantly higher than that of the WFNS score, mFisher score and serum AIM2 levels alone (all P < 0.05).

## Discussion

To the best of our knowledge, it is unclear regarding circulating levels of AIM2 in aSAH. In this study, serum AIM2 levels of aSAH patients were significantly higher than those of healthy controls. Alternatively, in clinical practice, WFNS and mFisher scores were conventionally utilized to evaluate disease severity, and predict functional outcome and DCI after aSAH. Currently, serum AIM2 levels were significantly positively correlated with mFisher scores and WFNS scores. Also, besides WFNS scores and mFisher scores, serum AIM2 levels were independently related to the occurrence of DCI and poor prognosis 90 days after aSAH. In addition, the predictive power of serum AIM2 levels for DCI and poor prognosis resembled those of mFisher scores and WFNS scores; moreover, the combination model, in which serum AIM2, WFNS scores and mFisher scores, displayed higher predictive efficiency for DIC or poor prognosis. To sum up, the increase of serum AIM2 level may be closely related to the severity of aSAH, and be independently related to functional prognosis and DCI, suggesting that serum AIM2 may be a potential biomarker for the prognosis of aSAH.

DCI, one of the most common and severe complications of aSAH, is the major cause of morbidity and mortality in patients who survive primary treatment of ruptured intracranial aneurysms^24^. The pathological processes of DCI involve cerebrovascular spasm, microcirculation contraction, microthrombosis, diffuse cortical ischemia and delayed apoptosis, which are all related to inflammation and oxidative stress after aSAH^25–27^. On the other hand, inflammation is the pivotal mechanisms, which are implicated in early brain injury following aSAH^28^. So, some biomarkers, which are pertinent to inflammation, may be promising predictive indicators of functional outcomes and DCI.

AIM2 is a member of the interferon-induced HIN-200 family of proteins and is a DNA receptor^29^. It is mainly localized in the cytoplasm and plays an important role in a number of inflammation-associated diseases^30^. AIM2 forms a functional inflammatory vesicle in cortical neurons, which is responsible for sensing intracellular damage caused by exogenous or endogenous sources^29,30^. It can recognize exogenous dsDNA and induce cystatinase-1 activation and release of mature inteuleukin-1beta^31^. So, AIM2 inflammasomes may be activated specifically in response to DNA damage. In the central nervous system, there are high expressions of AIM2 inflammasomal proteins, which may be implicated in cell death in neurons^32^.

In AIM2 knockout mice with vascular dementia, interleukin-1beta and -18 productions were significantly diminished and cognitive impairment was improved greatly^17^. Also, knockout of AIM2 obviously lessened blood–brain barrier destruction in mice after cerebral vascular occlusion^18,19^. Similarly, AIM2 knockout markedly reduced neuronal pyroptosis after subarachnoid hemorrhage and therefore decreased neurological impairment of rats^20^. AIM2 may be involved in damaged blood–brain barrier after traumatic brain injury^33^. Hence, it is believably accepted that AIM2 may be a detrimental factor in acute brain injury. AIM2 could be tremendously generated from brain tissues, mainly in glial cells after brain injury^34^. In our study, a portion of patients offered continuous blood drawings. These patients had similar demographic, clinical and biochemical data, as compared to the total patient population, indicating that these patients should represent the whole cohort of patients with aSAH. Noteworthily, our study showed that serum AIM2 levels in patients were significantly higher than those in healthy controls, and this increase lasted until at least 10 days after the onset of stroke. Therefore, it is postulated that blood AIM2 levels may be actually elevated after aSAH and moreover, AIM2 in blood may be at least partially derived from the damaged brain tissue after aSAH.

In the current study, as opposed to serum AIM2 levels at admission, those at days 1, 2, 3, 5, 7 and 10 had similar DCI and prognosis predictive abilities in terms of AUC. For the sake of investigating relationship between serum AIM2 levels and illness severity in addition to DCI and prognosis, serum AIM2 levels at admission were analyzed. Using multivariate analysis, serum AIM2 levels were independently correlated with WFNS score and mFisher score; moreover, the levels independently predicted DCI and poor prognosis. In our study, linear correlations and subgroup analyses were implemented. Presumably, serum AIM2 levels were linearly correlated with risks of DCI and poor prognosis. In addition, no interactions were found between serum AIM2 levels and other conventional variables, such as age, gender, hypertension and alcohol consumption. Overall, the preceding results are strongly supportive of the notion that serum AIM2 at admission may serve as a potential prognostic biochemical marker of aSAH.

As for the predictive capability, serum AIM2 levels efficiently differentiated between patients with DCI and those without, as well as between patients with poor prognosis and those with good prognosis. Intriguingly, the predictive values of serum AIM2 levels for the two prognostic indicators resemble those of WFNS score and mFisher score. Furthermore, the combination of serum AIM2 levels with WFNS score and mFisher score as a model, showed significantly improved prognostic ability, as compared with WFNS score and mFisher score alone. The two models were graphically represented. Theoretical calibration curves and decision curves were then created and subsequently it was verified that the models manifested well, with high stability and clinical benefits. Hence, it should be admitted that determination of serum AIM2 may assist to increase clinical prognostic ability of aSAH.

This study has certain limitations. First, we measured the serum AIM2 levels of patients at multiple time points and compared them with those of healthy volunteers, finding that AIM2 may have scientific value for the prognosis of aSAH and DCI patients. However, we acknowledge that the number of patients agreeing to undergo multiple-time-point blood-collection was limited, so this conclusion needs to be validated in studies with a larger sample size. Second, the prognosis and DCI of aSAH are closely related to the overall condition of the body^35^. aSAH not only damages brain tissue but also triggers systemic oxidative stress and inflammatory reactions^36^. AIM2 can promote the production of inflammatory factors interleukin-1β and interleukin-18 and cause intracellular damage when specifically activated^37^. Therefore, AIM2 may not only potentially harm the central nervous system but also have potential effects on the peripheral system^38^. Current research indicates that serum AIM2 levels are closely related to the 90-day prognosis and DCI of aSAH, and serum AIM2 may also be a biological marker that accurately reflects systemic inflammatory responses, but this requires further research for validation.

## Conclusions

To the best of our knowledge, this is a first series for unveiling the prognostic role of serum AIM2 in aSAH. Our main findings are that serum AIM2 levels are independently related to hemorrhage severity, DCI and 90-day functional outcome after aSAH; moreover, serum AIM2 exhibits significantly effective predictive ability for DCI and poor prognosis at 90 days following aSAH. Taken together, serum AIM2 may be a potential biomarker for assessing hemorrhage severity and predicting DCI and 90-day functional outcome after aSAH.

## Data Availability

The datasets generated and/or analyzed during the current study are not publicly available because they are personal data, but are available from the corresponding author upon reasonable request.

## References

[1] van Gijn J, Kerr RS, Rinkel GJ (2007). Subarachnoid hemorrhage. Lancet.

[2] Lauzier DC, Jayaraman K, Yuan JY, Diwan D, Vellimana AK, Osbun JW (2023). Early brain injury after subarachnoid hemorrhage: Incidence and mechanisms. Stroke.

[3] Topkoru B, Egemen E, Solaroglu I, Zhang JH (2017). Early brain injury or vasospasm? An overview of common mechanisms. Curr. Drug Targets.

[4] Macdonald RL (2014). Delayed neurological deterioration after subarachnoid hemorrhage. Nat. Rev. Neurol..

[5] Wang L, Zhou H, Zheng W, Wang H, Wang Z, Dong X (2024). Clinical value of serum complement component 1q levels in the prognostic analysis of aneurysmal subarachnoid hemorrhage: A prospective cohort study. Front. Neurol..

[6] Yan T, Chen Z, Zou S, Wang Z, Du Q, Yu W (2023). A prospective cohort study on serum A20 as a prognostic biomarker of aneurysmal subarachnoid hemorrhage. World J. Emerg. Med..

[7] Wu X, Ji D, Wang Z, Yu W, Du Q, Hu W (2023). Elevated serum NOX2 levels contribute to delayed cerebral ischemia and a poor prognosis after aneurysmal subarachnoid hemorrhage: A prospective cohort study. Neuropsychiatr. Dis. Treat..

[8] Zheng YK, Dong XQ, Du Q, Wang H, Yang DB, Zhu Q (2017). Comparison of plasma copeptin and multiple biomarkers for assessing prognosis of patients with aneurysmal subarachnoid hemorrhage. Clin. Chim. Acta.

[9] Xu Z, Kombe Kombe AJ, Deng S, Zhang H, Wu S, Ruan J (2024). NLRP inflammasomes in health and disease. Mol. Biomed..

[10] Hornung V, Ablasser A, Charrel-Dennis M, Bauernfeind F, Horvath G, Caffrey DR (2009). AIM2 recognizes cytosolic dsDNA and forms a caspase-1-activating inflammasome with ASC. Nature.

[11] Man SM, Kanneganti TD (2015). Regulation of inflammasome activation. Immunol. Rev..

[12] Man SM, Karki R, Kanneganti TD (2016). AIM2 inflammasome in infection, cancer, and autoimmunity: Role in DNA sensing, inflammation, and innate immunity. Eur. J. Immunol..

[13] Choubey D (2019). Type I interferon (IFN)-inducible absent in melanoma 2 proteins in neuroinflammation: Implications for Alzheimer's disease. J. Neuroinflammation.

[14] Chou WC, Guo Z, Guo H, Chen L, Zhang G, Liang K (2021). AIM2 in regulatory T cells restrains autoimmune diseases. Nature.

[15] Denes A, Coutts G, Lénárt N, Cruickshank SM, Pelegrin P, Skinner J (2015). AIM2 and NLRC4 inflammasomes contribute with ASC to acute brain injury independently of NLRP3. Proc. Natl. Acad. Sci. USA.

[16] Zheng Y, Tang W, Zeng H, Peng Y, Yu X, Yan F (2022). Probenecid-blocked pannexin-1 channel protects against early brain injury via inhibiting neuronal AIM2 inflammasome activation after subarachnoid hemorrhage. Front. Neurol..

[17] Poh L, Fann DY, Wong P, Lim HM, Foo SL, Kang SW (2021). AIM2 inflammasome mediates hallmark neuropathological alterations and cognitive impairment in a mouse model of vascular dementia. Mol. Psychiatry.

[18] Kim H, Seo JS, Lee SY, Ha KT, Choi BT, Shin YI (2020). AIM2 inflammasome contributes to brain injury and chronic post-stroke cognitive impairment in mice. Brain Behav. Immun..

[19] Franke M, Bieber M, Kraft P, Weber ANR, Stoll G, Schuhmann MK (2021). The NLRP3 inflammasome drives inflammation in ischemia/reperfusion injury after transient middle cerebral artery occlusion in mice. Brain Behav. Immun..

[20] Yuan B, Zhou XM, You ZQ, Xu WD, Fan JM, Chen SJ (2020). Inhibition of AIM2 inflammasome activation alleviates GSDMD-induced pyroptosis in early brain injury after subarachnoid hemorrhage. Cell Death Dis..

[21] Wang Q, Yu D, Liang J, Cheng Q, Zhou F, Lin H (2021). Significance of expression of AIM2, IL-1β, and IL-18 in plasma of patients with acute cerebral infarction. Zhong Nan Da Xue Xue Bao Yi Xue Ban.

[22] Vergouwen MD, Vermeulen M, van Gijn J, Rinkel GJ, Wijdicks EF, Muizelaar JP (2010). Definition of delayed cerebral ischemia after aneurysmal subarachnoid hemorrhage as an outcome event in clinical trials and observational studies: Proposal of a multidisciplinary research group. Stroke.

[23] Nobels-Janssen E, Postma EN, Abma IL, van Dijk JMC, Haeren R, Schenck H (2022). Inter-method reliability of the modified Rankin Scale in patients with subarachnoid hemorrhage. J. Neurol..

[24] Budohoski KP, Guilfoyle M, Helmy A, Huuskonen T, Czosnyka M, Kirollos R (2014). The pathophysiology and treatment of delayed cerebral ischaemia following subarachnoid hemorrhage. J. Neurol. Neurosurg. Psychiatry.

[25] Suzuki H, Kanamaru H, Kawakita F, Asada R, Fujimoto M, Shiba M (2021). Cerebrovascular pathophysiology of delayed cerebral ischemia after aneurysmal subarachnoid hemorrhage. Histol. Histopathol..

[26] Dorhout Mees SM, Kerr RS, Rinkel GJ, Algra A, Molyneux AJ (2012). Occurrence and impact of delayed cerebral ischemia after coiling and after clipping in the International Subarachnoid Aneurysm Trial (ISAT). J. Neurol..

[27] Rosenthal ES, Biswal S, Zafar SF, O'Connor KL, Bechek S, Shenoy AV (2018). Continuous electroencephalography predicts delayed cerebral ischemia after subarachnoid hemorrhage: A prospective study of diagnostic accuracy. Ann. Neurol..

[28] Weiland J, Beez A, Westermaier T, Kunze E, Sirén AL, Lilla N (2021). Neuroprotective Strategies in aneurysmal subarachnoid hemorrhage (aSAH). Int. J. Mol. Sci..

[29] Adamczak SE, de Rivero Vaccari JP, Dale G, Brand FJ, Nonner D, Bullock MR (2014). Pyroptotic neuronal cell death mediated by the AIM2 inflammasome. J. Cereb. Blood Flow Metab..

[30] Song X, Ma F, Herrup K (2019). Accumulation of cytoplasmic DNA due to ATM deficiency activates the microglial viral response system with neurotoxic consequences. J. Neurosci..

[31] Lugrin J, Martinon F (2018). The AIM2 inflammasome: Sensor of pathogens and cellular perturbations. Immunol. Rev..

[32] Hu B, Jin C, Li HB, Tong J, Ouyang X, Cetinbas NM (2016). The DNA-sensing AIM2 inflammasome controls radiation-induced cell death and tissue injury. Science.

[33] Ge X, Li W, Huang S, Yin Z, Xu X, Chen F (2018). The pathological role of NLRs and AIM2 inflammasome-mediated pyroptosis in damaged blood-brain barrier after traumatic brain injury. Brain Res..

[34] Barclay WE, Aggarwal N, Deerhake ME, Inoue M, Nonaka T, Nozaki K (2022). The AIM2 inflammasome is activated in astrocytes during the late phase of EAE. JCI Insight.

[35] Li X, Zeng L, Lu X, Chen K, Yu M, Wang B (2023). Early brain injury and neuroprotective treatment after aneurysmal subarachnoid hemorrhage: A literature review. Brain Sci..

[36] Chai CZ, Ho UC, Kuo LT (2023). Systemic inflammation after aneurysmal subarachnoid hemorrhage. Int. J. Mol. Sci..

[37] Fan Z, Chen R, Yin W, Xie X, Wang S, Hao C (2023). Effects of AIM2 and IFI16 on infectious diseases and inflammation. Viral Immunol..

[38] Sharma M, de Alba E (2021). Structure, activation and regulation of NLRP3 and AIM2 inflammasomes. Int. J. Mol. Sci..

